# Standardizing SPECT/CT dosimetry following radioembolization with yttrium-90 microspheres

**DOI:** 10.1186/s40658-021-00413-3

**Published:** 2021-10-30

**Authors:** S. Peter Kim, Daniel Juneau, Claire Cohalan, Shirin A. Enger

**Affiliations:** 1 Medical Physics Unit, McGill University, Montreal, Canada; 2 Biological and Biomedical Engineering, McGill University, Montreal, Canada; 3 Department of Medical Imaging, Centre Hospitalier de l’Université de Montréal, Montreal, Canada; 4 Department of Physics and Biomedical Engineering, Centre Hospitalier de l’Université de Montréal, Montreal, Canada; 5 Lady Davis Institute for Medical Research, Jewish General Hospital, Montreal, Canada

**Keywords:** Dosimetry, SPECT/CT, Local deposition method, Self-calibrations, Tissue mass densities, Radioembolization, SIRT

## Abstract

**Background:**

Multiple post-treatment dosimetry methods are currently under investigation for Yttrium-90 ($^{90}\hbox {Y}$) radioembolization. Within each methodology, a variety of dosimetric inputs exists that affect the final dose estimates. Understanding their effects is essential to facilitating proper dose analysis and crucial in the eventual standardization of radioembolization dosimetry. The purpose of this study is to investigate the dose differences due to different self-calibrations and mass density assignments in the non-compartmental and local deposition methods. A practical mean correction method was introduced that permits dosimetry in images where the quality is compromised by patient motion and partial volume effects.

**Methods:**

Twenty-one patients underwent $^{90}\hbox {Y}$ radioembolization and were imaged with SPECT/CT. Five different self-calibrations (FOV, Body, OAR, Liverlung, and Liver) were implemented and dosimetrically compared. The non-compartmental and local deposition method were used to perform dosimetry based on either nominal- or CT calibration-based mass densities. A mean correction method was derived assuming homogeneous densities. Cumulative dose volume histograms, linear regressions, boxplots, and Bland Altman plots were utilized for analysis.

**Results:**

Up to 270% weighted dose difference was found between self-calibrations with mean dose differences up to 50 Gy in the liver and 23 Gy in the lungs. Between the local deposition and non-compartmental methods, the liver and lung had dose differences within 0.71 Gy and 20 Gy, respectively. The local deposition method’s nominal and CT calibration-based mass density implementations dosimetric metrics were within 1.4% in the liver and 24% in the lungs. The mean lung doses calculated with the CT method were shown to be inflated. The mean correction method demonstrated that the corrected mean doses were greater by up to $\sim 5$ Gy in the liver and lower by up to $\sim 12$ Gy in the lungs.

**Conclusions:**

The OAR calibration may be utilized as a potentially more accurate and precise self-calibration. The non-compartmental method was found more comparable to the local deposition method in organs that were more homogeneous in mass densities. Due to the potential for inflated lung mean doses, the non-compartmental and local deposition method implemented with nominal mass densities is recommended for more consistent dosimetric results. If patient motion and partial volume effects are present in the liver, our practical correction method will calculate more representative doses in images suboptimal for dosimetry.

## Background

Three-dimensional voxel-based dosimetry is an active area of investigation in an internal radiation therapy called radioembolization to improve upon its simple clinical dosimetry. Radioembolization is an angiographic procedure where a catheter is used to guide and inject Yttrium-90 ($^{90}\hbox {Y}$) microspheres into a specifically selected hepatic artery. Due to unique hepatic blood flow, the microspheres are distributed preferentially in tumor arteries, where they are permanently deposited. The tumor is then irradiated by the beta particles emitted from $^{90}\hbox {Y}$ decay.

Radioembolization’s dosimetry may be grouped into two categories that include methods clinically practiced and those researched within the field. The dosimetric methodologies clinically suggested are the partition and the non-compartmental models [1, 2]. These models are based on the Medical Internal Radiation Dose (MIRD) schema and apply two gross assumptions; (1) the injected microsphere sources distribute uniformly within entire regions of interest (ROI), which may include an entire organ and (2) the administered activity is contained entirely within the liver and lungs [2]. The clinical methodologies typically utilizes planar gamma imaging to calculate a lung shunt fraction as well as to perform simple dosimetry. Significant limitations to planar gamma imaging exist due to a lack of proper attenuation, scatter, and volume corrections. In contrast, radioembolization’s voxel-based methods are directly image-based and combine functional PET or SPECT images containing information regarding positions of the injected radionuclides with anatomical CT images to produce a 3D activity distribution. For SPECT/CT, traditionally qualitative reconstructions have significantly progressed to produce algorithms that are quantitative in nature [3–6]. Quantitative SPECT/CT images enable patient-specific treatment planning and subsequent dose verification.

### Current status of $^{90}\hbox {Y}$ dosimetry

Methodologies for $^{90}\hbox {Y}$ SPECT/CT dosimetry have become more established in recent years. Before dosimetric maps are produced, a quantitative SPECT image that depicts hardware-specific counts must first be converted to activity in each voxel. Since $^{90}\hbox {Y}$ is deposited directly into the hepatic vasculature, it is assumed that a single abdominal FOV contains all relevant activity. The image can be self-calibrated by relating the SPECT counts in the FOV with the total injected activity [7–12]. However, there is no consensus regarding the exact volume within the FOV in which it is assumed all true counts are contained.

The clinical non-compartmental and partition methods have implicitly assumed that the relevant counts are contained solely within the liver and the lung [2]. Within voxel-based methods, self-calibrations have included the liver itself, the patient’s body, and the entire SPECT FOV [7–12]. A study comparing these calibrations have indicated large dosimetric variations, but the current clinical assumptions were not addressed [13]. There is also a possibility that microspheres may shunt extrahepatically to organs such as the gallbladder, stomach, duodenum, kidneys, and lungs, despite coil embolization [14–16]. A new calibration that includes the most likely areas of extrahepatic depositions may be implemented and explored.

Post-treatment dosimetry may be performed once quantitatively reconstructed counts are converted to activity. Previous studies have demonstrated that the spatial resolution of SPECT systems is the main limitation to accurate dosimetry [9, 17]. When comparing between voxel-based methods, all methodologies have shown comparable dosimetric accuracies [9, 10, 17]. In particular, the ease of use and potentially superior accuracy of the local deposition method has made it a favored dosimetry model [18]. The non-compartmental and partition methods used clinically may also be implemented for post-treatment dosimetry when voxel-based methods are unavailable. The comparability between the local deposition and these clinical methods is still unknown for $^{90}\hbox {Y}$ SPECT/CT. Moreover, the local deposition method can be implemented in different ways by utilizing either nominal- or CT-based mass densities [10, 12, 13, 18]. Nominal densities assume that densities within organs are homogeneous while CT-derived densities use a scanner-specific Hounsfield unit to density calibration curve. Due to a lack of consensus regarding mass calculations in the manufacturer guidelines, either mass density implementation may be further applied to the clinical methodologies. The dose effects between mass density implementations have not been investigated for the local deposition method nor the clinical methodologies.

Patient motion and partial volume effects are other factors that affect dosimetric accuracy [9, 10, 19]. Image blurring is unavoidable in SPECT/CT dosimetry. However, patient motion or partial volume effects that spread activities outside organ delineations cause the dosimetric validity on such images, especially when activities bleed between organs, to become questionable. A dosimetric correction method would be useful in addressing these imaging limitations.

The purpose of this study was to aid in the standardization of radioembolization’s $^{90}\hbox {Y}$ SPECT/CT dosimetry. To achieve this goal, this study had the following aims: (1) to expand upon the dosimetric effects of utilizing new and existing self-calibrations, (2) compare the dosimetry in mass density implementations of the non-compartmental and local deposition method, and (3) demonstrate a practical correction method to account for patient motion and partial volume effects.

## Materials and methods

### Patient population

This retrospective study received approval from our institutional review board and was performed on an anonymized patient-based cohort of 21 $^{90}\hbox {Y}$ SPECT/CT image sets. All patients were treated with glass microspheres (TheraSphere; Boston Scientific, USA) and clinical dosimetry was planned using the non-compartmental method. The mean injected activity was $3.01 \pm 2.05$ GBq with a range of 0.649–8.96 GBq. The injected activity for each patient was calculated based on tumor burden, liver volume, and lung shunting was taken into consideration. Two patient cohorts were categorized within this study: optimal and suboptimal. Table 1 lists a summary of all variables tested. Inclusion criteria for the optimal cohort were images that allowed for accurate organ contours on low-dose CT, counts that were completely delineated within CT contours (i.e., no motion artifacts nor excessive partial volume effects) for optimal dose comparisons, and complete dosimetry information. Only five patients remained for optimal local deposition comparisons. Six patients had CT images that were unable to be contoured due to insufficient contrast, two patients lacked sufficient treatment information to perform dosimetry, and eight patients had counts outside CT contoured delineations that were deemed from patient motion or partial volume effects. These same eight patients were categorized as the suboptimal cohort. The optimal cohort was used to test variables 1–7 while the suboptimal cohort was used to demonstrate our correction methodology. Truncated lungs within the FOV were not an exclusion criteria. Only one patient had their entire abdomen and lung visualized within the SPECT/CT FOV.

**Table 1:** List of dosimetric variables tested

Tested variable (Units)	Analysis method	Analysis result
1. Volumes (%)	$max(\frac{Nominal-CT}{CT},\frac{CT-Nominal}{Nominal})$	Volume bias
2. Interpolations (Distance)	Chi-square histogram	Interpolation bias
3. Self-calibrations	Linear regression, boxplots	Dose %s, dose difference
4. Mass densities	Linear regression slopes	Dose %s
5. Mass density means (Gy)	Bland Altman	95% CIs
6. Mass density DVH (Gy)	Bland Altman	95% CIs
7. NC vs. LDM methods (Gy)	Boxplots	Dose difference
8. Correction means (Gy)	Boxplots	Dose difference

*LDM* local deposition method

### Image acquisitions

All patient images were acquired on a Discovery NM/CT 670 SPECT/CT system (GE Healthcare, Cleveland, USA) with a parallel-hole medium-energy general purpose collimator. SPECT data were acquired with a 109.1–134.2 keV window for 120 views over $360^{\circ }$ with 30 s/view. Quantitative reconstructions were performed on HybridRecon (Version 1.3, Hermes Medical Solutions) and consisted of 3D ordered-subset expectation maximum (OSEM) reconstructions with attenuation correction based on a low-dose helical CT, scatter corrections based on a Monte Carlo convolution-based forced detection algorithm, and collimator detector response modeling [20]. All quantitative reconstructions were performed with an equivalent 75 iterations (15 subsets and 5 iterations) based on manufacturer recommendations [6]. No post-filter was applied and the reconstructed SPECT voxel sizes were isotropic at 4.42 mm.

When required, CT mass densities were calculated using a scanner-specific linear lookup table based on electron density phantom scans. The CT voxels were reconstructed with sizes 0.976 mm $\times$ 0.976 mm $\times$ 3.75 mm. All contours were drawn on the low-dose helical CT using tools from MIM Maestro v6.6 (MIM) and followed established contouring guidelines [21]. Any overlapping contours were corrected with Boolean operations. The contours were verified by an experienced nuclear medicine physician.

### pyreDose

pyreDose is an in-house open-sourced package developed for this study. It was used to perform image processing and dosimetry. pyreDose is written in Python and currently consists of dosimetry methods based on the local deposition method, the clinical methodologies, and their variations. pyreDose was used to process DICOM images, create self-calibration, perform interpolations, and calculate absorbed doses. To perform such analysis, each patient’s set of SPECT and CT images and contoured structures saved in DICOM RT format were imported to pyreDose.

### Self-calibrations

Prior to performing dosimetry, SPECT counts were converted to activity specific to a self-calibration. The self-calibration was implemented by equating the administered activity to the total counts within specified SPECT ROI to convert voxel counts into activity per patient.

The different self-calibrations were determined based on their clinical relevance and use within the literature [7–12]. They were titled FOV, Body, OAR, Liverlung, and Liver calibrations. The FOV calibration represented the assumption that all SPECT counts have valid activity. The Body calibration illustrates a conservative, but robust post-processing methodology based on MIM’s auto-body contouring [13]. Because microspheres cannot travel outside the body, all body contours were manually checked to remove any inclusion of contoured arms and corrected for any air pockets. The organs at risk (OAR) calibration was introduced to test a new calibration method. These organs included organs with likely microsphere depositions based on a standard hepatic vasculature. These organs were the liver, lung, gallbladder, stomach, proximal duodenum, and kidneys. The Liverlung calibration contained the liver and the lung counts and represented the clinical dosimetric assumptions. Although it is well documented that microspheres travel into the lungs, standalone liver calibrations have been increasingly used for dosimetry and imaging studies [4, 9, 13]. The Liver calibration was included to illustrate the effects of such an assumption.

### Image-based dosimetry

In this study, dosimetry was performed with two separate mass densities implementations for the non-compartmental (NC) method and the local deposition method [1, 2, 10, 22]. All absorbed doses were calculated based on $^{90}\hbox {Y}$ SPECT/CT images. If nominal-based densities were mapped for entire organs, it was titled the nominal method. If voxel-by-voxel density assignment was based on CT values, it was titled the CT method. For dosimetry, the CT method requires prepossessing so that the SPECT voxels match in size and position to the co-registered CT densities. To perform dosimetry without any loss of information, the SPECT image was interpolated to match the CT voxels using the nearest neighbor algorithm. This method was chosen among multiple interpolation methods since it performed best in a correspondence analysis as shown in Table 2.

**Table 2:** Preprocessing biases between the nominal and CT image inputs are summarized for organ volume calculations and interpolation methodologies

Patient	Volume bias (%)		Quadratic chi-histogram (Distance)			
	Liver	Lung	Nearest neighbor	Bilinear	Biquadratic spline	Bicubic spline
Patient 1	0.229	1.324	$\hbox {1.350}e10^{-5}$	$\hbox {3.677}e10^{-4}$	$\hbox {4.972}e10^{-3}$	$\hbox {5.275}e10^{-3}$
Patient 2	0.050	0.798	$\hbox {6.780}e10^{-5}$	$\hbox {1.728}e10^{-4}$	$\hbox {1.727}e10^{-3}$	$\hbox {1.863}e10^{-3}$
Patient 3	0.439	3.684	$\hbox {1.569}e10^{-5}$	$\hbox {3.340}e10^{-4}$	$\hbox {5.595}e10^{-3}$	$\hbox {5.917}e10^{-3}$
Patient 4	0.487	0.273	$\hbox {5.600}e10^{-5}$	$\hbox {2.080}e10^{-4}$	$\hbox {3.115}e10^{-3}$	$\hbox {3.316}e10^{-3}$
Patient 5	0.675	0.895	$\hbox {1.081}e10^{-5}$	$\hbox {3.204}e10^{-4}$	$\hbox {5.314}e10^{-3}$	$\hbox {5.640}e10^{-3}$

The local deposition method was implemented with a voxel dose as indicated in Eq. 1 with image voxel activities $x_{k}$ (GBq), mass densities $\rho _{k}$ ($\hbox {g/cm}^3$), voxel volumes $\Delta {V}_{k}$ ($\hbox {cm}^3$), and an activity to absorbed energy constant of 49.88 (J/GBq). The constant was calculated based on $^{90}\hbox {Y}$'s average β-energy per disintegration $E_{\text{avg}}$ (MeV/dis), its half-life $t_{1/2}$ (s), and a unit conversion variable u ((J*dis)/(MeV*s*GBq)).

1 $$\begin{aligned} \mathrm{Dose}_{k} = \frac{x_{k}}{\rho _{k}}*\frac{\overbrace{E_{\mathrm{avg}}*\frac{1}{\mathrm{ln}(2)}*t_{\frac{1}{2}}*u}^{\mathrm{const}}}{\Delta {V}_{k}} \end{aligned}$$

The NC method was calculated with the same parameters as Eq. 1, but following the NC assumptions where *n* in Eq. 2 refers to the total voxels within an organ.

2 $$\begin{aligned} \hbox {Dose}_{\mathrm{NC}} =\frac{\sum \nolimits _{k=1}^{n}x_{k}*{\mathrm{const}}}{\sum \nolimits _{k=1}^{n} \rho _{k}*\Delta {V}_{k}} \end{aligned}$$

The nominal local deposition and NC methods necessitate nominal densities based on ICRU report 46 to be mapped onto a SPECT image [23]. Density values of 1.06 $\hbox {g/cm}^3$ and 0.26 $\hbox {g/cm}^3$ were used as the respective liver and lung densities for $\rho _{k}$ while $x_{k}$ was based on the original SPECT image. The CT local deposition and NC methods utilized the mass densities $\rho _{k}$ derived from a CT calibration curve while $x_{k}$ was derived from interpolated SPECT images.

### Calculating corrected mean doses

Imaging issues such as patient motion and partial volume effects cause dose inaccuracies when valid voxel activities become spatially misplaced. Radioembolization has an abdominal FOV and activity in the liver commonly spills over into neighboring organs due to motion as scan times are long and free breathing is required. Partial volume effects may further cause activities within the liver to bleed into the lung [10]. A potential correction may be implemented if the initial spatial locations of such activities are known, but typically this is not the case. By applying the assumptions of the local deposition method and assuming homogeneous/nominal mass densities, a theoretical relocation of counts to the correct voxel is possible without requiring a priori knowledge of its spatial location. A practical mean correction method may be derived starting with the equation of the mean dose, the assumption of homogeneous mass densities $\rho$ and equal voxel volumes $\Delta {V}$.

3 $$\begin{aligned} \hbox {Dose}_{\mathrm{corr}} = \frac{\mathrm{const}}{n*p*\Delta {V}} * \left( \sum _{k=1}^{n}x_{k}+\sum _{j=1}^{n_{\mathrm{misplaced}}}y_{j} \right) \end{aligned}$$

**Fig. 1:**
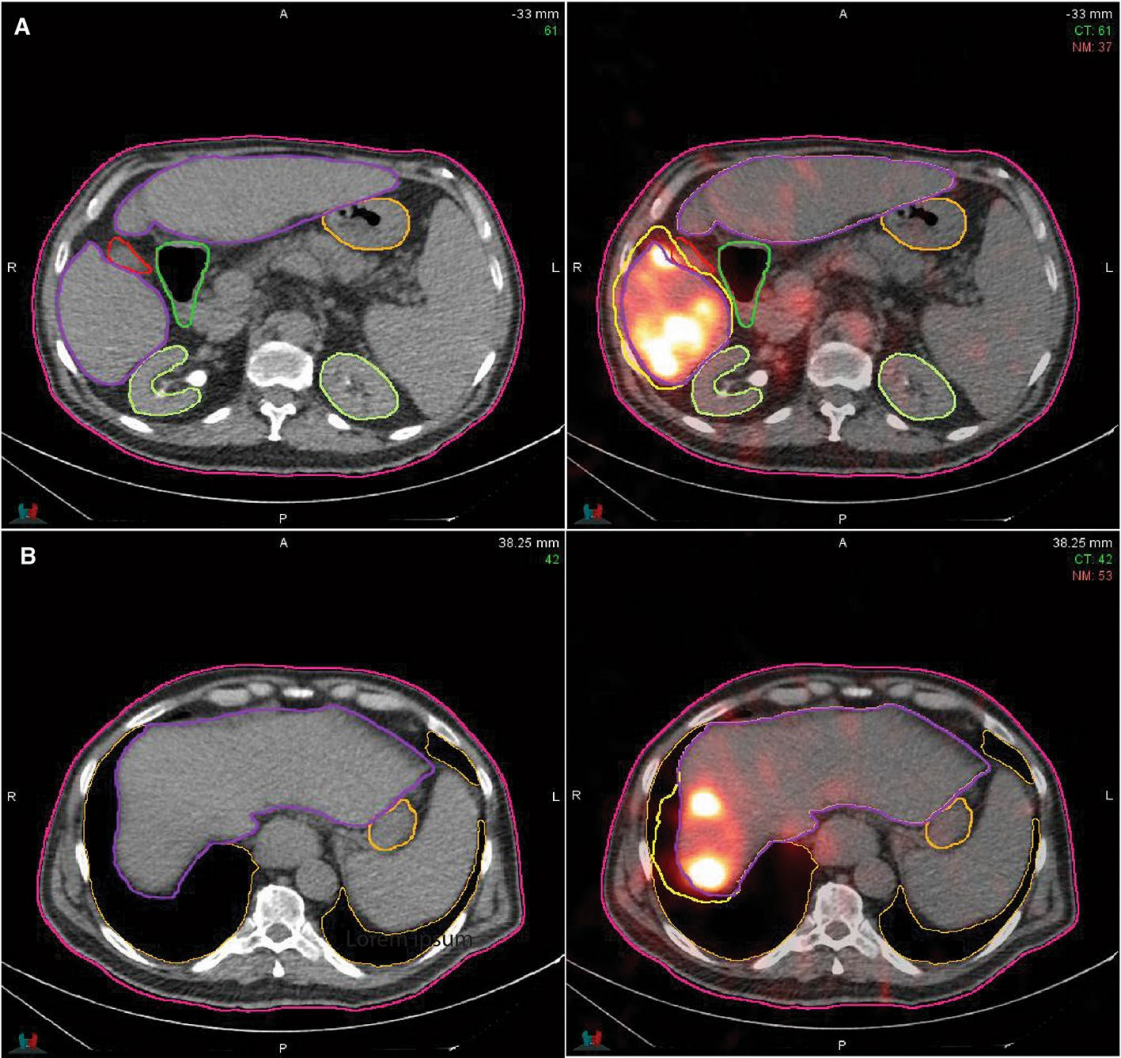
Contour of two axial slices for one patient are visualized where the left represents the CT and the right represents a SPECT/CT-fused image. The purple contour represents the original liver contours whereas the yellow represents the correction contour that includes valid, but misplaced activities either due to patient motion or partial volume effects

Equation 3 displays the derived correction method to calculate mean organ absorbed doses with the original activities $x_{k}$ and the addition of misplaced activities $y_{j}$. The correction method was used to obtain more representative liver mean doses when activities in the liver bled into other organs. These corrections were achieved by creating correction contours that included counts outside organ delineations. Illustrated in Fig. 1, $y_{j}$ was defined as counts contained within the corrected volume, but outside the original contours. If any counts spilled into other organs such as the lungs, the same counts were excluded from the other organs. Because self-calibrations are dependent on organ delineations, the corrected organ contours were utilized to create corrected self-calibrations before dosimetry.

### Statistical tests

All statistical tests were performed and visualized with Python. The quadratic chi-histogram distance metric was used to compare the error between interpolation methods as seen in Eq. 4.

4 $$\begin{aligned} \hbox {distance} = \frac{1}{2} \sum _{i=1}^{n} \frac{(a_{i}-b_{i})^2}{a_{i}+b_{i}} \end{aligned}$$

This test measures the image correspondence between two differing images and their normalized histograms with the same binning. $a_{i}$ and $b_{i}$ represent the two frequency histograms and their respective bins *i*. A lower distance metric pertains to closer correspondence (i.e., a more accurate interpolation) where a distance of 0 represents equated histograms. Cumulative dose volume histograms (DVHs) were computed and $D_{x}$ was defined as the minimum dose that x% volume would obtain. Bland Altman analysis was performed to illustrate the dosimetric differences between local deposition methodologies and correction differences. Multiple DVH dose metrics ($D_{70}$, $D_{50}$, $D_{30}$, $D_{10}$), mean dose estimates, and all self-calibrations were used to calculate the mean, standard deviation, and 95% confidence intervals (CI) of bias for the Bland Altman analyses. If heteroscedastic trends were observed in any Bland Altman plots, the same data were $\hbox {log}_{10}$ transformed, then used to recalculate their CIs and means biases that now represented log-ratios [24]. When creating Bland Altman plots, any log-transformed CIs were then back-transformed and plotted on the regular scale for intuitive visual analysis [25]. These back-transformed slopes were defined as CIs that are proportional to the *x*-axis mean values of the Bland Altman plots. When performing linear regressions, the same dosimetric metrics were compared between the CT and nominal local deposition methods. The correlation coefficients (*r*) and their slopes were computed.

## Results

### Image processing discrepancies

Table 2 summarizes the discrepancies in the image inputs between the nominal and CT data. The nominal methods utilize contours drawn on CT and overlaid to the original SPECT images, which may result in organ volume errors. The liver and lung volume differences between the CT and nominal inputs resulted in only a maximum volume deviation of 3.7%. The CT methods require interpolations that have their own biases. The nearest neighbor interpolation consistently performed the best with the smallest similarity distances between the interpolated and original quantitative SPECT images.

### Self-calibration comparisons

**Fig. 2:**
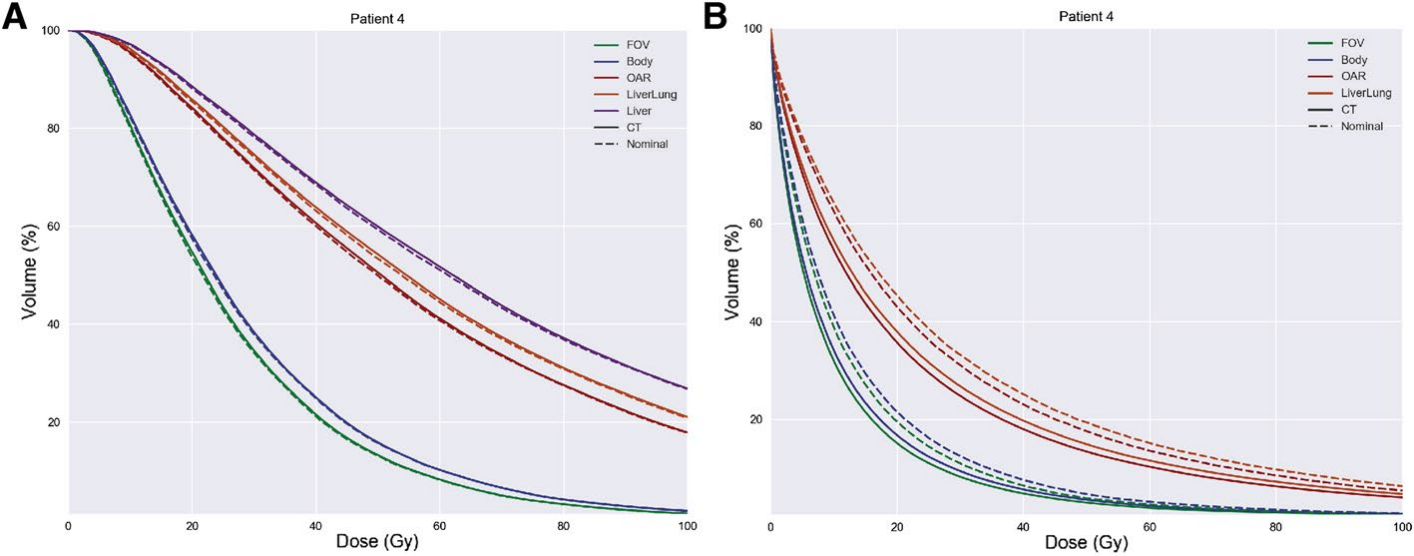
Liver (**A**) and lung (**B**) cumulative dose volume histograms (DVHs) are shown for one patient. Each calibration includes the DVH calculated using the CT and nominal local deposition methods

A patient’s liver and lung DVHs are shown for the local deposition method within Fig. 2. Visually, the FOV and Body calibrations and the OAR, Liverlung, Liver calibrations were grouped together to create two calibration groupings. The liver DVHs between the CT and nominal local deposition methods were visually indistinguishable from one another. Overall as the calibrations included smaller ROI, the DVHs shifted right and upward.

The mean doses of multiple self-calibrations were compared through linear regression slopes, which are summarized within Table 3. Correlation coefficients (*r*) were > 0.958 between all mean doses of varying self-calibrations, illustrating excellent linear correlations. These linear regression slopes represent a weighted dose factor and, consequently, a percentage change that occurs between mean doses of varying calibrations. In this context, the liver had an 8.8% difference in the calculated dose between the FOV and Body calibrations, and a $\sim 200\%$ difference between the Body and OAR calibrations. There was a $\sim 15\%$ difference between the liver mean doses calculated with the Liverlung and the Liver calibrations. When compared to the FOV calibration, both the liver and lung mean dose factors increased as the calibrations changed from the Body to the OAR and finally to the Liverlung. Within the liver, the FOV and Liver calibrations had the highest percentage differences at $\sim 270\%$ for both the nominal and CT local deposition methods. For the lung, the FOV and Liverlung comparisons had the highest percentage differences ranging from 230% to 240% for the CT and nominal local deposition methods.

**Table 3:** The CT and nominal local deposition methods were compared between their respective calibrations for both the liver and lungs. These comparisons were performed on their mean doses and within the same mass density method. The calibration column represents the x-axis of the linear regression where the bold represents a regression slope between the same calibrated data

Region	Calibration	Linear regression slopes									
		FOV		Body		OAR		Liverlung		Liver	
		CT	Nominal	CT	Nominal	CT	Nominal	CT	Nominal	CT	Nominal
Liver	FOV	**1.000**	**1.000**	1.081	1.088	2.239	2.254	2.361	2.374	2.712	2.729
	Body	0.923	0.916	**1.000**	**1.000**	2.069	2.067	2.180	2.173	2.502	2.498
	OAR	0.446	0.443	0.482	0.483	**1.000**	**1.000**	1.054	1.051	1.210	1.209
	Liverlung	0.423	0.421	0.457	0.459	0.948	0.951	**1.000**	**1.000**	1.149	1.151
	Liver	0.361	0.366	0.398	0.398	0.823	0.825	0.870	0.869	**1.000**	**1.000**
Lung	FOV	**1.000**	**1.000**	1.100	1.069	2.275	2.210	2.382	2.329	X	X
	Body	0.907	0.933	**1.000**	**1.000**	2.065	2.063	2.162	2.170	X	X
	OAR	0.439	0.451	0.483	0.483	**1.000**	**1.000**	1.046	1.053	X	X
	Liverlung	0.419	0.428	0.462	0.457	0.955	0.949	**1.000**	**1.000**	X	X

**Fig. 3:**
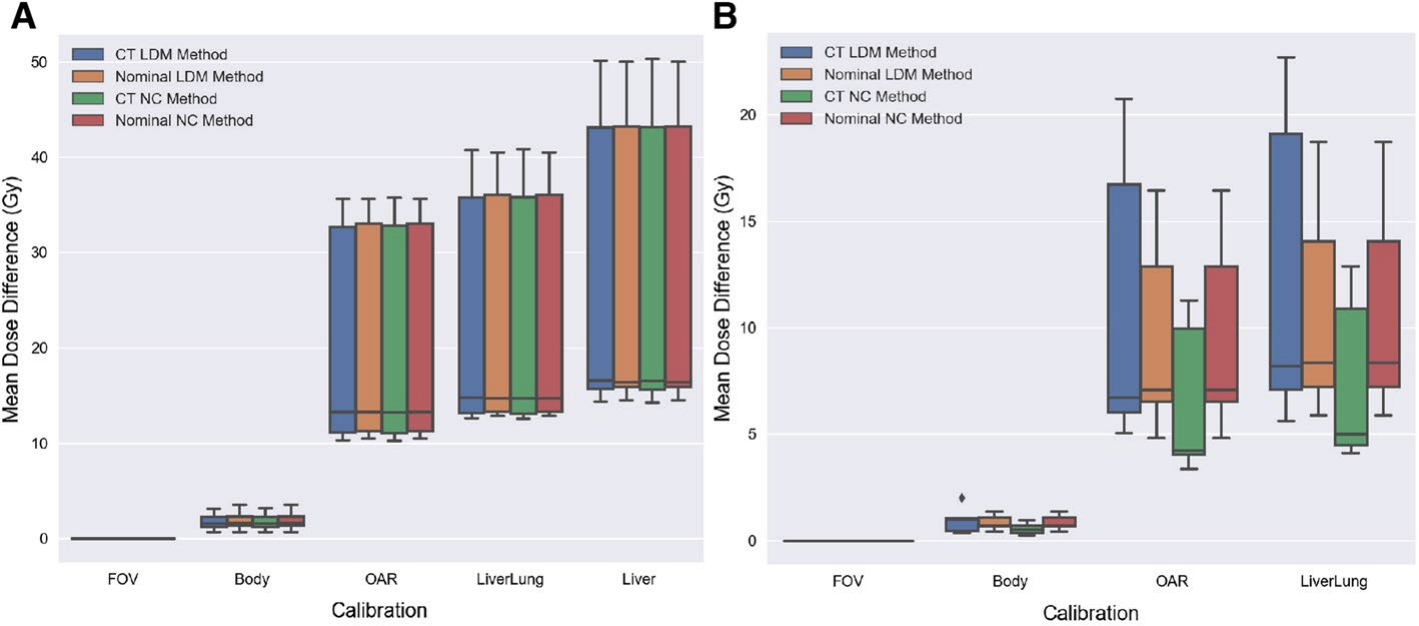
Liver (**A**) and lung (**B**) absolute mean dose differences of each methodology relative to their own FOV calibration. The absolute mean dose difference between calibrations may be calculated by subtracting the calibration mean dose differences from one another

Figure 3 summarizes the absolute mean dose differences of different calibrations relative to the FOV calibration within each dosimetric methodology. The total ranges of dose differences within each calibration are 0.68–3.54 (Gy), 10.3–35.8 (Gy), 12.6–40.9 (Gy), and 14.3–50.3 (Gy) for the Body, OAR, Liverlung, and Liver calibrations, respectively. For the lungs, the total ranges of dose differences are 0.37–2.0 (Gy), 4.01–20.8 (Gy), and 4.13–22.6 (Gy) for the Body, OAR, and Liverlung calibrations, respectively.

### Nominal vs. CT local deposition methods

**Fig. 4:**
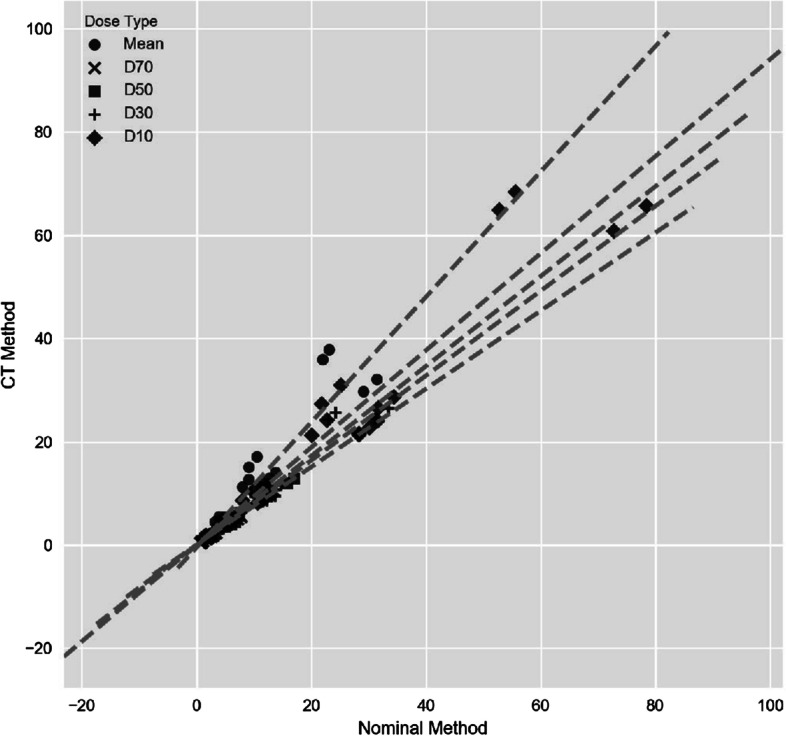
The linear regressions represent weighted dose factors that compares the mass density differences between the CT and nominal local deposition methods for a particular dose metric, $N=20$. *N* represents the number of data points compared for each dose metric for the lungs

The difference in each dose metric ($D_{70}$, $D_{50}$, $D_{30}$, $D_{10}$, and mean) between the CT and nominal local deposition methods was investigated with linear regressions, which are shown in Fig. 4. Table 4 lists the linear regression slopes and their corresponding *r* values. The regression slopes between the CT and nominal local deposition methods demonstrated excellent correlations with all their *r* $\ge$ 0.91. Table 4 demonstrates that the CT local deposition method had all its liver dose metrics within 1.4% of the doses calculated with the nominal local deposition method. For the lung, most regression slopes were below 1.0 indicating most dose estimates for the CT local deposition method were generally lower than those of the nominal method. There was one exception; the lung mean doses were generally greater with a 21.3% increase for the CT method over the nominal method.

**Table 4:** Linear regression slopes between the CT and nominal local deposition method was summarized for multiple dose metrics

Metric	Liver		Lung	
	Slope	*r* value	Slope	*r* value
Mean	1.005	0.999	1.213	0.910
$D_{70}$	1.013	0.999	0.756	0.981
$D_{50}$	1.014	0.999	0.821	0.972
$D_{30}$	1.003	0.999	0.870	0.966
$D_{10}$	1.001	0.999	0.940	0.942

**Fig. 5:**
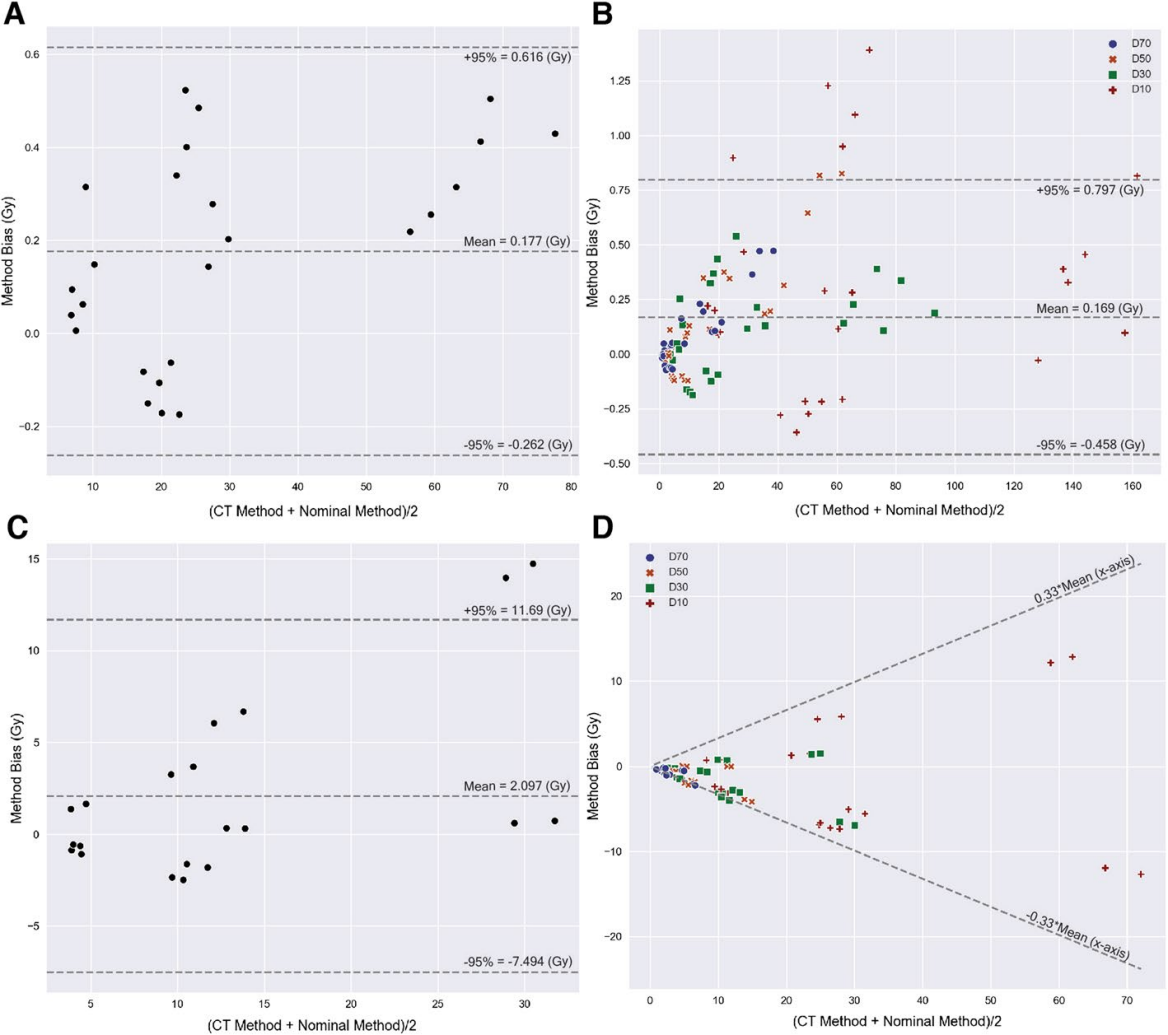
The Bland Altman analysis plots the mean bias and 95% CIs between the CT and nominal local deposition methods for the mean doses (**A**, **C**) and all the cumulative dose volume histogram (DVH) dose metrics (**B**, **D**). Parts **A**, **B** refer to the liver while **C**, **D** refer to the lungs. Parts **A**, **C** compare between every self-calibration mean dose estimate for the liver $N=25$ and the lungs, $N=20$. Parts **B**, **D** compare between every self-calibration DVH dose metric in the liver $N=100$ and the lungs $N=80$. *N* represents the number of data points compared for each Bland Altman plot. The lungs did not include the Liver calibration

Figure 5A, C represents the overall absorbed dose variability between the mean doses for every calibration of the CT and nominal local deposition methods. The bias was defined as the mean dose of the CT method subtracted by the mean dose of the nominal method. The liver mean bias was 0.18 Gy while the lung mean bias was 2.1 Gy suggesting larger mean liver and lung doses for the CT local deposition method. Similarly, Fig. 5B, D represents the overall absorbed dose variability between all DVH dose metrics for every calibration of the CT and nominal deposition methods. Its bias was defined as the DVH dose metric of the CT method subtracted by the corresponding DVH dose metric of the nominal method. The mean bias of the liver DVH dose metrics was 0.17 Gy, indicating the DVH doses based on the CT method are slightly greater than those of the nominal method. In contrast, the mean log-ratio bias of the lung DVH was − 0.1, demonstrating that the lung DVH dose metrics based on the nominal method are generally greater than the CT method. The back-transformed slope was 0.33, showing that the 95% CI for lung dose biases is within 33% of the *x*-axis mean values.

### Non-compartmental dose comparisons

**Fig. 6:**
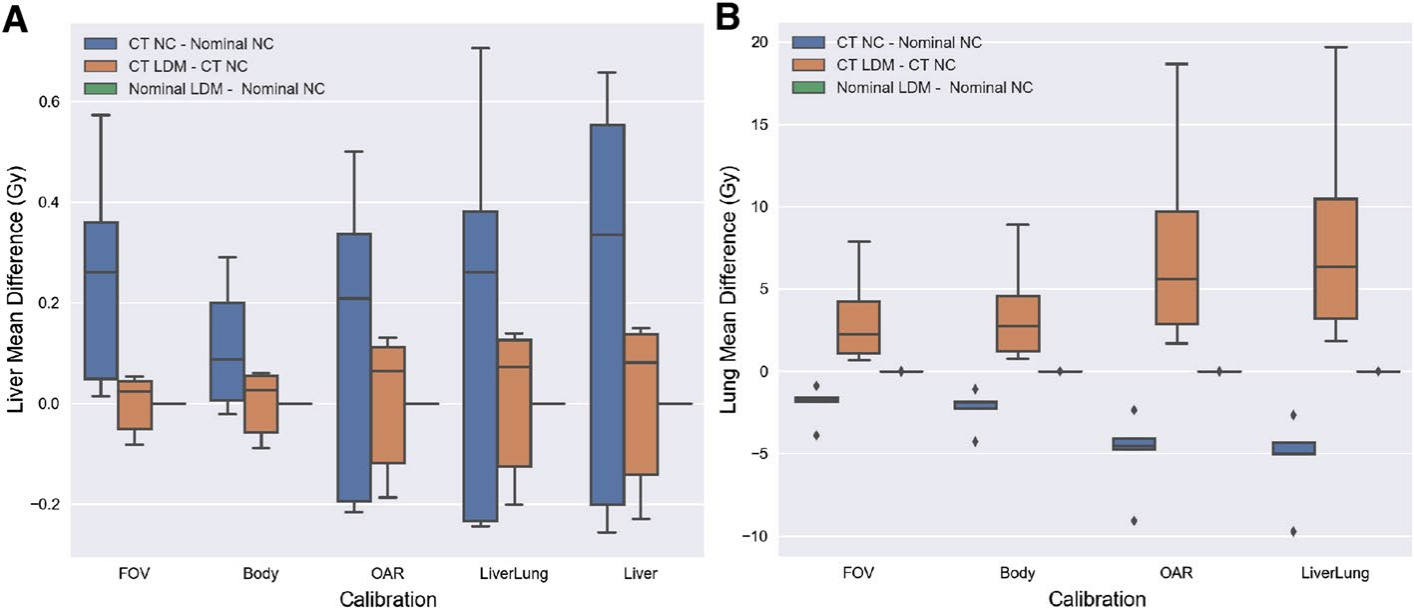
A boxplot of the mean dose differences between the CT and nominal implementations of the NC and local deposition methods (LDM) for the (**A)** liver and (**B)** lung. The legend summarizes the calculation performed

Figure 6 illustrates the dosimetric comparisons between consistently calibrated methodologies. Between the CT and nominal NC methods, the Liver doses were within 0.71 Gy while the lung doses were within 10 Gy. The comparisons between the CT methods of the NC and local deposition methods resulted in differences within 0.23 Gy for the liver and within 20 Gy for the lungs. However, all the dose differences between the nominal NC and nominal local deposition methods were 0 Gy.

### Corrected mean doses

**Fig. 7:**
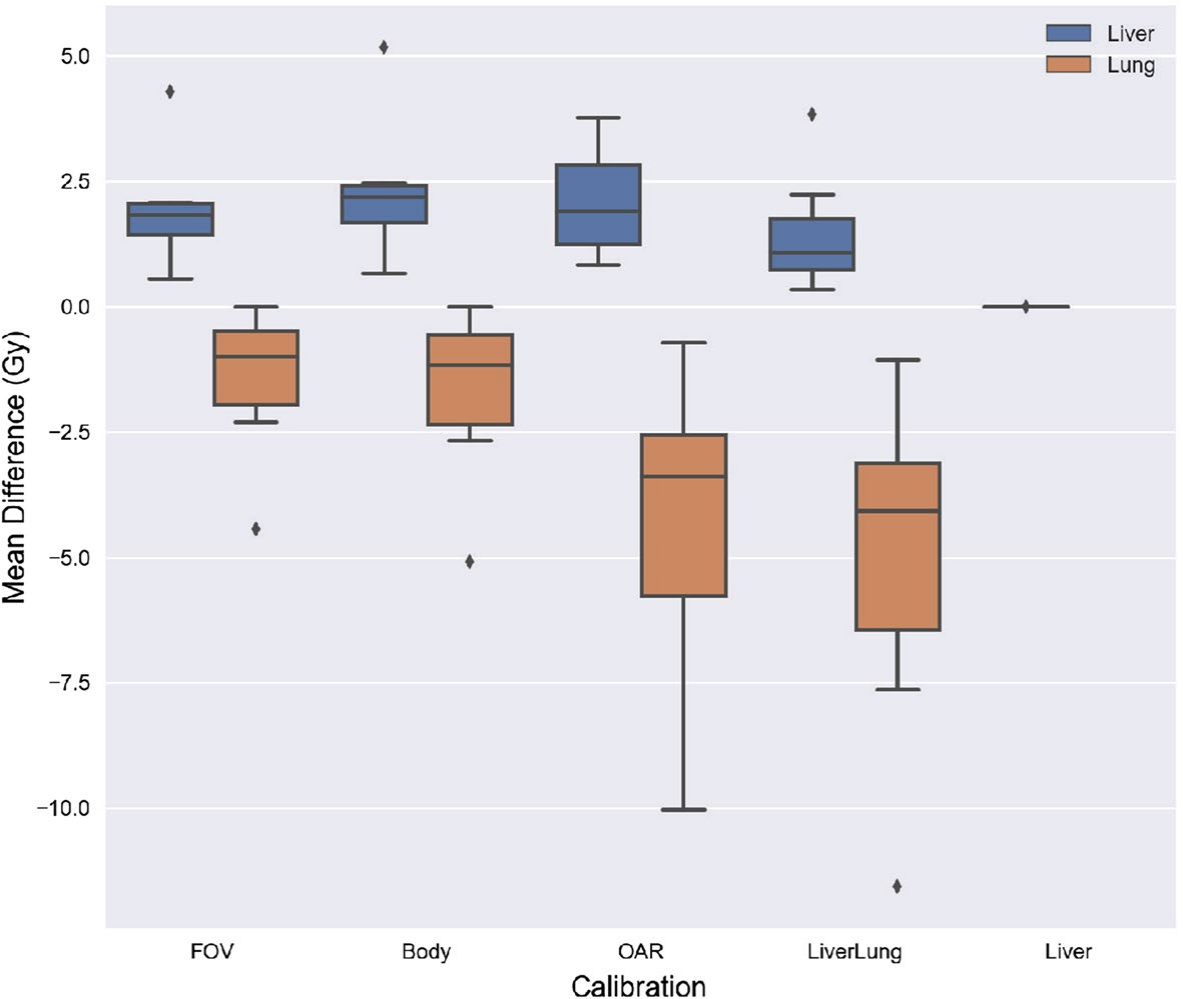
A boxplot of corrected mean doses for both the liver, $N=40$ and lungs, $N=32$. *N* represents the total number of data points compared for each particular organ

The liver tends to lose activities, which are typically gained in the lungs. This results in mean dose corrections where the absolute lower limit for the liver and absolute upper limit for the lung are both 0 Gy. Therefore, these correction differences were combined and illustrated in Fig. 7. As shown in Fig. 7 the corrected mean doses stayed relatively consistent ($< 5.5$ Gy) while the corrected lung doses showed a trend where smaller calibration regions lead to increasingly decreased lung dose estimates ($< 12.5$ Gy). All eight of the liver mean differences based on the liver calibration were 0 Gy.

## Discussion

This study investigated the absorbed dose differences between several dosimetry variables calculated with the NC and local deposition methods. The NC method is a dosimetric method that was heuristically created due to technological limitations. Commonly performed on planar imaging, the NC method calculated on SPECT/CT will have improved dosimetric accuracy. On the other hand, the local deposition method has been empirically validated for SPECT/CT, but neither validation studyhas incorporated the different self-calibration and mass density implementations in their methodologies [9, 18]. Additional studies are required to investigate the accuracy of each dosimetry variable implementation. However, empirical conclusions require phantom measurements that cannot yet be directly translated to clinical situations. Consequently, this study based on real patient data is essential for proper dosimetric interpretation and comparability. Our results demonstrate that even within the same methodology there is a considerable dose variability when the dosimetry inputs are inconsistent. These implementation variations are applicable concerns in all dosimetric methodologies from the NC method to Monte Carlo simulations. These results emphasize the need for standardization for reliable dosimetry comparisons.

### Self-calibrations

The greatest dose differences were obtained between the different self-calibration implementations. Our largest dose differences ranged up to 50 Gy for the liver and 23 Gy for the lungs (Fig. 3). When comparing our results with previously reported values, our mean dose differences were much larger with a linear regression slope of $\sim 2.7$ compared to their slope of $\sim 1.7$ between the FOV and Liver calibrations [13]. These dosimetric differences were most likely attributed to reconstruction and patient FOV differences.

With large dose differences between calibrations, a conclusion on the most appropriate self-calibration would be helpful. Definite conclusions are difficult because SPECT images contain a combination of noise, scattering, collimator errors, as well as the correct count data. To accurately choose the proper self-calibration, the spatial distribution of $^{90}\hbox {Y}$ microspheres must be known. This information requires identifying the microsphere depositions within a patient’s microvasculature, which is not readily achieved [15].

Instead, standardizing the use of self-calibrations remains the more pressing concern for comparable dose analysis (Table 3). In this regard, certain calibrations may be non-ideal for implementation. In general, microspheres flow indiscriminately and may even shunt into extrahepatic arteries after coil embolization [15, 26]. Thus, the Liver calibration should not be used. In the same vein, the FOV calibration is precluded because it includes counts that are outside a patient’s body and cannot be attributed to the injected microspheres.

In our study, the microsphere depositions most plausibly predominated in the liver and lungs. Our patient cohort was properly coiled, had no extrahepatic and radiation induced side-effects, nor had any activity depositions visible outside the liver and lungs on SPECT/CT. Based on such patient characteristics, the Liverlung calibration was likely the most accurate self-calibration for our patient cohort. Interestingly, there exists a dramatic mean dose difference of $\sim 220\%$ between the Body and Liverlung calibration, but when the OAR was compared to the Liverlung calibration only a maximum of 5.4% (Table 3) or 5.1 Gy underestimation resulted even after the addition of four organs (Fig. 3). These results indicate that if any additional calibration organs were added, the mean dose differences will be comparable to the OAR rather than the Body calibration. This further suggests the dose discrepancy between the Liverlung and OAR calibration is attributed to reconstruction errors and noise, likely caused by imaging bremsstrahlung photons. If due to reconstruction effects, the OAR’s 5.4% underestimation may be clinically acceptable by greatly improving dosimetric precision without sacrificing accuracy should any significant depositions occur within any common organs at risk. For the Body calibration, its large volume likely includes many erroneous counts greatly reducing its accuracy [13]. Calibrating based on all organs with suspected microsphere deposition similar or equal to the OAR calibration may potentially be the ideal calibration methodology.

### Mass density differences

The SPECT/CT interpolations demonstrated near perfect correspondence to the original quantitative image regardless of its interpolation method. Minimal interpolation differences coupled with low volume differences demonstrate simple preprocessing steps are adequate for SPECT/CT dosimetry. Consequently, the estimated dose differences between the CT and nominal methods for both the NC and local deposition methods were found to be predominately caused by mass density effects.

As shown in Table 4 and Fig. 5, all liver dose metrics were comparable regardless of their mass density implementation. The assumption that the liver is a homogeneous organ was empirically confirmed. For the lungs, Table 4 demonstrates lower DVH percentage differences at smaller volumes ($D_{30},D_{10}$). Although percentage differences were lower, Fig. 5B, D still showed large mean dose biases at smaller volumes. These results demonstrate that lung dose estimates between the nominal and CT methods are highly variable.

Both Fig. 4 and Table 4 suggest lung mean doses are generally much greater for the CT method than the nominal method. As opposed to the mean doses, the lung DVH dose metrics were shown to be generally greater for the nominal method at all the same volume coverages as the CT method ($D_{70},D_{50},D_{30},D_{10}<1.0$ ). These results are contradictory because greater DVH dose metrics for the nominal method should produce correspondingly greater mean doses. This contradiction was found to be caused by the large distribution of lung densities within the CT method; more specifically, many voxels were identified with mass densities close to air. If any signal, however small, is interpreted as activity and converted to energy where such low density voxels are localized, these voxels would result in highly inflated absorbed doses. As shown in Fig. 2, these inflated dose estimates explain the rightward shift for the CT method at lower $D_{x}$ volumes. In other words, the CT local deposition method’s lung dose metrics, especially mean and lower volume $D_{x}$ dose estimates, are sensitive to stray counts (i.e., noise, scatter, system blur) and should be interpreted with caution. The nominal mass densities will likely provide more consistent dose estimates for lung dosimetry.

### Non-compartmental dose comparisons

Figure 6 highlights the comparability between the NC and local deposition methods when image inputs such as modality type, self-calibrations, and mass densities are consistent. Mean dose differences between these methodologies were found to be largely attributable to differences in mass densities where organs with more homogeneous densities provided more comparable results. Figure 6 illustrated such effects when the dose bias spread between the CT local deposition and CT NC method was smaller for the liver than the lungs. The lung mean doses were also found to be consistently greater for the CT local deposition method when compared to the CT NC method. This can be attributed to the differences between the local deposition and NC calculations where the NC method homogeneously spreads the total energy deposited over its total mass. This spreading of energy deflates the overall mean dose when compared to the local deposition method where individual energy depositions are normalized by its voxel mass. Between the CT methods, the local deposition method will likely result in greater mean doses for organs with heterogeneous densities. Furthermore, Eqs. 1 and 2 make apparent that the NC and local deposition methods will result in the same dose estimates when implementing nominal densities. This suggests that as long as nominal mass densities and consistent quantitative reconstructions are utilized, the dose estimates from the non-compartmental and extended to the partition method will lead to comparable mean dose estimates to voxel-based methodologies.

### Dosimetric corrections

Our correction method may be used to obtain more representative mean doses for suboptimal images when dosimetry may not otherwise be possible. Although an essential component for dosimetry analysis, most, if not all studies do not separate between optimal and suboptimal patient datasets. In this study, the mean doses were corrected for images where activities in the liver spilled over into neighboring organs. Figure 7 showed that the magnitude of the mean dose corrections for the lungs were greater than the livers’. This was explained by the lung’s relatively smaller fraction of activity with respect to the total included activity counts, resulting in a greater dosimetric shift with minimal correction changes. In contrast, the liver mean doses stayed relatively consistent between calibrations.

Eight mean liver doses calibrated with the Liver rationale were shown to have a dose difference of 0 Gy. This result highlights an important consequence of using self-calibrations; they implicitly ignore counts outside organ delineations even when they are the product of patient motion and partial volume effects. For accurate dosimetry, any misplaced counts must be included during calibration because they still contribute to the relative activity distributions within an image. If not included, resulting calibrated activities will have activity distributions that are off by a dosimetric factor.

Importantly, this correction methodology has an empirically valid basis when correcting for the liver. Our results have shown that the liver doses have minimal differences between density implementations. Studies have shown that the liver doses calculated with the local deposition method are at worst comparable and at best superior to other voxel-based methodologies [9, 10, 17, 18]. Consequently, the correction doses will be comparable to the mean doses calculated by other voxel-based methodologies. In fact, because all liver activities have been accounted for, voxel-based dosimetry may still be performed in other organs. To note, a potential limitation exists. If all blurred activities due to patient motion or partial volume effects are accounted for, the local deposition method has the potential to overestimate the liver’s mean doses [9]. However, Fig. 7 demonstrates that if an overestimation does occur it will likely be negligible due to the liver’s relatively large activity before correction. Nonetheless, our mean correction method will permit more accurate dosimetry in other organs such as the lungs where doses were shown to be more affected by misplaced activities.

### Limitations

Owing to the retrospective nature of this study, there were several limitations. SPECT/CT FOVs were not standardized, which resulted in patients having varying lung volume cut-offs. As with all voxel-based studies, these results were specific to one set of optimized reconstruction parameters, which limit the applicability of these results. Monte Carlo-based collimator corrections that increase reconstruction accuracy have been developed, but are not commonly available [6, 27]. This study did not implement such corrections. Rather, a more feasible, but still quantitative reconstruction was implemented. Lastly, this study was performed on a small number of ideal patients. These results will need to be confirmed with a well-designed prospective study and a statistically meaningful number of patients.

## Conclusion

This study investigated the dosimetry differences in preprocessing, self-calibration, and mass density implementations between the non-compartmental and local deposition methods. A mean correction method was further introduced. There was a large dose heterogeneity, up to 50 Gy in liver and 23 Gy in lungs when self-calibrations and mass density implementations were varied. This corresponded to up to a $\sim 270\%$ difference between calibration implementations. Mass density comparisons between the nominal and CT method found consistent doses for the liver, but was highly variable in the lungs. Between the non-compartmental and local deposition method, mean doses were more comparable in organs with homogeneous densities; in fact, mean organ doses were the same between the two methods when nominal densities were utilized. Overall, standardization of radioembolization’s dosimetry must be achieved for comparable dosimetric analysis to be viable in the future. In this regard, the implementation of the OAR calibration and nominal mass densities may provide the most ideal methodology for standardization. If patient motion effects and partial volume effects are present in the liver, our mean correction method will calculate more representative mean doses.

## Data Availability

The datasets generated during and/or analyzed during the current study and pyreDose software are available from the corresponding author on reasonable request.
